# Global Bi-ventricular endocardial distribution of activation rate during long duration ventricular fibrillation in normal and heart failure canines

**DOI:** 10.1186/s12872-017-0530-5

**Published:** 2017-04-13

**Authors:** Qingzhi Luo, Qi Jin, Ning Zhang, Yanxin Han, Yilong Wang, Shangwei Huang, Changjian Lin, Tianyou Ling, Kang Chen, Wenqi Pan, Liqun Wu

**Affiliations:** Department of Cardiology, Shanghai Ruijin Hospital, Shanghai Jiao Tong University School of Medicine, No. 197, Ruijin Er Road, Shanghai, 200025 People’s Republic of China

**Keywords:** Ventricular Fibrillation, Activation Rate, Heart Failure

## Abstract

**Background:**

The objective of this study was to detect differences in the distribution of the left and right ventricle (LV & RV) activation rate (AR) during short-duration ventricular fibrillation (SDVF, <1 min) and long-duration ventricular fibrillation VF (LDVF, >1 min) in normal and heart failure (HF) canine hearts.

**Methods:**

Ventricular fibrillation (VF) was electrically induced in six healthy dogs (control group) and six dogs with right ventricular pacing-induced congestive HF (HF group). Two 64-electrode basket catheters deployed in the LV and RV were used for global endocardium electrical mapping. The AR of VF was estimated by fast Fourier transform analysis from each electrode.

**Results:**

In the control group, the LV was activated faster than the RV in the first 20 s, after which there was no detectable difference in the AR between them. When analyzing the distribution of the AR within the bi-ventricles at 3 min of LDVF, the posterior LV was activated fastest, while the anterior was slowest. In the HF group, a detectable AR gradient existed between the two ventricles within 3 min of VF, with the LV activating more quickly than the RV. When analyzing the distribution of the AR within the bi-ventricles at 3 min of LDVF, the septum of the LV was activated fastest, while the anterior was activated slowest.

**Conclusions:**

A global bi-ventricular endocardial AR gradient existed within the first 20 s of VF but disappeared in the LDVF in healthy hearts. However, the AR gradient was always observed in both SDVF and LDVF in HF hearts. The findings of this study suggest that LDVF in HF hearts can be maintained differently from normal hearts, which accordingly should lead to the development of different management strategies for LDVF resuscitation.

## Background

Frequency analysis using the fast Fourier transform (FFT) has been widely used to characterize features of ventricular fibrillation (VF). Electrical and optical mapping experiments in previous animal and human studies have evaluated the spatiotemporal distribution of the activation rate (AR) during VF and have provided mechanistic insight into the organization of VF, thereby improving our understanding of the initiation and maintenance of this complex arrhythmia [1–7]. The regional frequency characteristics may be related to a fixed anatomic myocardial substrate and dynamic physiological factors such as refractory periods [8]. It has also been postulated that the fastest activating region drives fibrillation throughout the rest of the myocardium by giving rise to activation fronts that propagate into the more slowly activating regions.

The activation of VF changes as it continues, which raises the possibility that the relative importance of different arrhythmogenic mechanisms changes. It has been proposed that defibrillation mechanisms and efficacy may differ in different pathological animal models during different stages of VF [9]. Panfilov et al. reported that the excitation frequency is an important index in VF that can reflect the underlying myocardial pathophysiology; it may also be predictive of the defibrillation threshold [10]. Previous studies have reported that the dynamics of VF in heart failure (HF) hearts differ from those in normal hearts, with a substantial decrease in AR [3, 11]. The aim of this study was to determine the distribution of AR across two fibrillating global ventricular endocardium samples at different stages in normal and HF canine hearts. We hypothesized that as the duration of VF continued, there would be quantifiable regional AR varieties in the inter-ventricles and/(or) intra-ventricles.

## Methods

### Pacemaker implantation

Twelve beagles (11 ± 1.2 kg) were divided into two groups. Six dogs were selected to create the HF model, and the other six dogs served as the control group. A pacemaker (Kappa 710, Medtronic, Minneapolis, Minnesota, USA) was implanted in a subcutaneous pocket and attached to a pacing lead (5076, Medtronic, Minneapolis, Minnesota, USA) in the RV apex under fluoroscopic visualization via the right external jugular vein. When the surgery was completed, the dogs were given antibiotics, after which they underwent rapid ventricular pacing at 240 beats per minute for three to 4 weeks. Then, echocardiography was performed to confirm that the HF model was successfully established. Rapid pacing was maintained until a day before the electrophysiological mapping study.

### Animal preparation

**Fig. 1:**
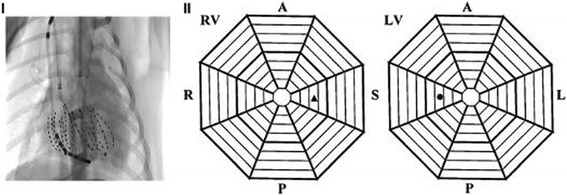
Global electrical mapping of the RV and LV endocardium. Panel I shows a fluoroscopic image of a posterior–anterior view of two basket catheters in the LV and RV and the RV defibrillation catheter. Panel II shows the basket orientation in the LV and RV. R, right free wall; A, anterior free wall; L, left free wall; P, posterior free wall; and S, septum. Apical electrodes are placed toward the center of the display, and basal electrodes are located near the periphery

Each animal was injected intramuscularly with ketamine (10 mg/kg) and tropine (0.04 mg/kg) for anesthetic induction. Anesthesia was maintained intravenously with propofol (8–16 mg/kg/h), and the animals were ventilated in a restrained, dorsally recumbent position. To determine the adequacy of the anesthesia, ventilation and oxygenation, the arterial blood pressure, blood gases, cardiac electrical activity, body temperature and serum electrolytes were monitored, and interdigital reflexes were tested throughout the entire study. After the completion of the data collection, the animals were euthanized with an intravenous injection of potassium chloride. The hearts were exposed through a median sternotomy and supported in a pericardial sling. A catheter (model 80,993, IBI, St. Jude Medical, Saint Paul, Minnesota, USA) was inserted for defibrillation with the negative electrode in the RV apex and the positive electrode in the superior vena cava (Fig. 1).

### Bi-ventricular endocardial mapping

A multielectrode basket (Constellation Catheter, model US8031U, Boston Scientific, Natick, MA, USA) was introduced through the left carotid artery into the LV. Another basket catheter was inserted through the right jugular vein into the RV. Each catheter contained eight splines each with eight (8 × 8) electrodes approximately 2 mm apart. These two catheters were used to map the ventricular endocardium simultaneously (Fig. 1). A detailed description of this technique was presented in our earlier report [9].

### VF induction and AR analysis

VF was induced by a 30 Hz stimulation delivered (MicroPace III, EPS 320 Cardiac stimulator) through one of the basket electrodes. Four VF episodes were induced in each animal. The first three VF episodes were recorded for 20 s as a short-duration (SDVF) episode before being halted by a 400–600 V biphasic shock (6/4 ms) delivered from a defibrillator electrode. The last VF episode was allowed to continue for at least 3 min (long-duration VF, LDVF), and the animal was not resuscitated. The first 20 s of VF data during this LDVF episode served as the fourth SDVF episode. There were no significant differences in the VF parameters among multiple short VF episodes. Additionally, the AR distribution did not depend on the basket electrodes used to induce VF. The two 64-electrodes of the basket catheter and the six limb-ECG leads were recorded with a 160-channel cardiac data acquisition system. The AR was estimated through FFT analysis of VF at each electrode of the basket catheter and the six limb leads of the body ECG for a 2 s interval beginning 20 s after VF induction. The frequency with the highest power between 1 and 20 Hz was taken as the AR.

### Statistical analysis

To ascertain how the AR in different ventricular regions varied during VF, the endocardium of the two ventricles were divided into 8 zones according the display of the basket electrode (Fig. 1). We defined the lower half of the basket as the apex and the upper half as the base. Data are given as the mean ± SD. Analysis of variance was used to test for significant differences among the mean AR of the zones for the VF episodes, followed by a Fisher’s protected least significance difference to determine which zones differed significantly. To determine the difference between the apex and the base of the RV, LV in AR during VF, the data were analyzed for significance using a paired *t* test. A value of *P*<0.05 was considered significant.

## Results

### HF model

There was unambiguous evidence (tachypnea, lethargy and ascites) of myocardial systolic dysfunction in the HF group but not in the control animals. The LV ejection fraction for the HF canines was substantially decreased (63 ± 5.3% vs. 29.5 ± 8.2%, *P* < 0.0001), accompanied by significant increases in the LV end-diastolic dimension and LV end-systolic dimension.

### Regional distribution of VF AR in the control group

**Fig. 2:**
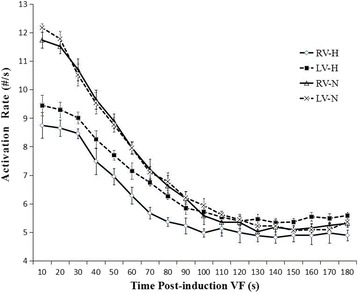
Evolution of the AR during VF. In the control group, there was only an AR gradient between the ventricles within the first 20 s. In contrast, in the HF group, there was a detectable bi-ventricular AR gradient for the entire VF duration. From 90 s to the end of analysis at 3 min of VF, neither the RV or LV in the HF hearts activated differently than those of the control animals. (LV-N: left ventricle in the normal group; LV-H: left ventricle in the HF group; RV-N: right ventricle in the normal group; RV-H: right ventricle in the HF group.) See the text of the article for additional details

**Fig. 3:**
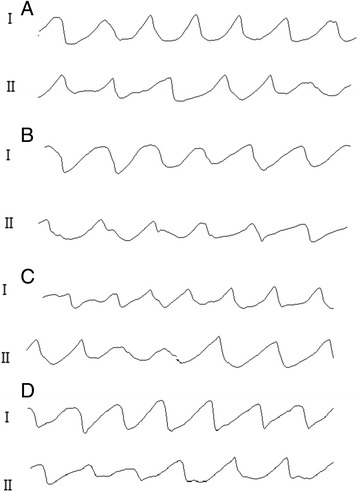
Snapshots of activation during VF in one normal and one HF-affected dog at the 3 min LDVF. Recordings of VF activation from one control heart are shown in Fig. 3 **a** and **b**, with Fig. 3 **a** I representing the posterior wall and II representing the anterior wall of the LV. In Fig. 3 **b**, I indicates the posterior wall, and II indicates the anterior wall of RV; as shown, the posterior wall activates faster than the anterior wall. Recordings of VF activation from one HF-affected animal are shown in Fig. 3 **c** and **d**. Figure 3 **c** I shows the septal wall, and II shows the anterior wall of the LV. Figure 3 **d** I shows the septal wall, and II indicates the anterior wall of the RV; the septal wall activates faster than the anterior wall

**Fig. 4:**
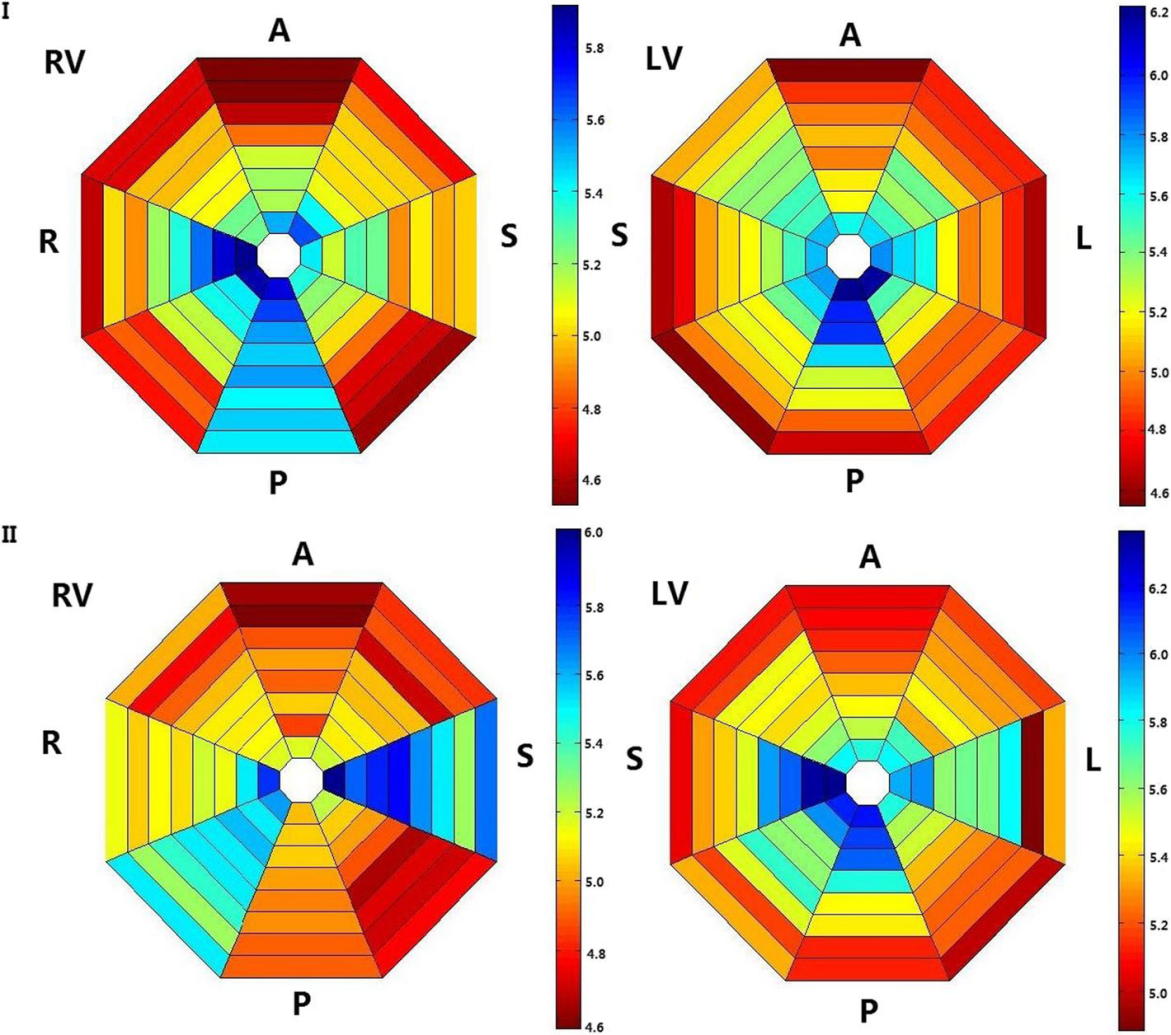
3 min LDVF regional AR distribution in the RV and LV in one normal and one HF animal. Figure 4 I shows the regional AR distribution of the two ventricles in the control group for the 3 min LDVF. In the LV, the posterior wall activates before the anterior wall. In the RV, the posterior wall also activates before the anterior wall. Figure 4 II represents the regional AR distribution of the RV and LV in the HF group for the 3 min LDVF. In the LV, the septal tissue activates the fastest, while the anterior wall activates the slowest. In the RV, the septal tissue activates the fastest, while the anterior wall activates the slowest. The colors represent the AR of the 64-basket electrodes according to the time scale shown to the right (blue represents the fastest activation and red represents the slowest activation)

**Fig. 5:**
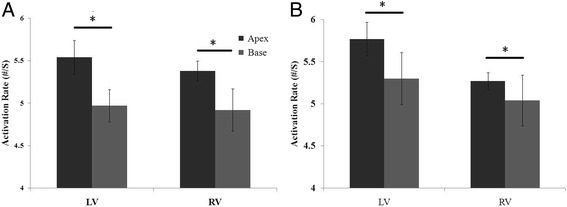
Three-minute LDVF AR distribution of the apical and basal portions of the bi-ventricles. Figure 5 **a** represents the apex-base AR differences in the control group, with the apex activating faster than the base in the LV (*P<0.01) and RV (*P<0.01). Figure 5 **b** shows the apex-base AR differences in the HF group, with the apex activating faster than the base in the LV (*P<0.01) and RV (*P<0.01)

In the control group, the LV activated faster than the RV (12 ± 0.3 vs. 11.6 ± 0.3, *P* = 0.04) in the first 20 s. However, there was no significant difference in the AR between LV and RV after 20 s. There was a dramatic decrease in AR between 20 s and 90 s, after which it declined smoothly. At 3 min, there was no significant difference between the LV and RV (5.23 ± 0.20 vs. 5.27 ± 0.11, *P* = 0.27) (Fig. 2). When analyzing the distribution of AR within LV, the posterior wall of the LV activated the fastest (I in Fig. 3a), while the anterior wall was the slowest (II in Fig. 3a), with a 7% difference between the fastest and the slowest activating regions (5.50 ± 0.54 vs. 5.09 ± 0.24, *P* = 0.024). When analyzing the distribution of AR within the RV, the posterior wall activated the fastest (I in Fig. 3b), while the anterior wall was the slowest (I in Fig. 3b), with an 11% difference between the fastest and the slowest activating regions (5.55 ± 0.13 vs. 4.95 ± 0.29, *P*<0.001) (Fig. 4). Additionally, the apical wall activated faster than the basal wall in both ventricles of the normal hearts with LV apex>LV base(5.54 ± 0.20 vs. 4.97 ± 0.12, *P*<0.01); and RV apex>RV base (5.38 ± 0.19 vs. 4.92 ± 0.25, *P*<0.01) (Fig. 5a).

### Regional distribution of the VF AR in the HF group

In the HF group, a detectable AR gradient always existed between the two ventricles, with the LV activating more quickly than the RV after VF induction. In the first 20 s interval, the LV activated faster than the RV (9.4 ± 1.15 vs. 8.7 ± 1.1, *P* = 0.02). At 3 min, the LV still activated more quickly than the RV (5.54 ± 0.76 vs. 5.08 ± 0.66, *P* = 0.004) (Fig. 2). When analyzing the distribution of AR within LV, the septum of the LV activated the fastest (I in Fig. 3c), while the anterior wall activated the slowest (II in Fig. 3c), with a 7% difference between the fastest and the slowest activating regions (5.75 ± 0.49 vs. 5.33 ± 0.25, *P* = 0.016). When analyzing the distribution of the AR within the RV, the septum of the RV was found to be activated the fastest (I in Fig. 3d), while the anterior wall was found to be activated more slowly (II in Fig. 3d), with an 11% difference between the fastest and the slowest activating regions (5.50 ± 0.11 vs. 4.88 ± 0.20, *P*<0.001) (Fig. 4). In addition, a significant AR gradient was observed between the apical and basal portions of both ventricles in the HF hearts, with LV apex>LV base (5.77 ± 0.20 vs 5.30 ± 0.10, *P*<0.01); and RV apex>RV base (5.27 ± 0.31 vs 5.04 ± 0.30, *P*<0.01) (Fig. 5b).

### Differences in the AR distribution between the normal and failing hearts

There were several differences between the two groups. First, relative to normal hearts, the AR of the HF group was much slower in the first 90 s, and a significant LV-RV AR gradient was always present. Second, at the first 20 s interval, there was a clear LV-RV AR gradient in the HF group relative to the normal canine hearts (0.66 ± 0.18 vs. 0.40 ± 0.22, *P* = 0.015). Additionally, the LV-RV AR gradient was greater at the beginning than at the end of the entire VF episode in the HF group (0.66 ± 0.18 vs. 0.45 ± 0.21, *P* = 0.009) (Fig. 2). Third, the activation of VF differed between the two groups at the 3 min LDVF. In the control group, the posterior of the LV activated fastest, while the anterior wall activated the slowest. In the HF group, the septum of the LV was activated the fastest, while the anterior wall was activated more slowly (Fig. 4).

## Discussion

The major findings of this study are as follows. (1) In the control group, the LV activated slightly faster than the RV in the first 20 s, after which there was no difference in the AR between the LV and RV. At 3 min of LDVF, the posterior of the LV activated fastest, while the anterior activated the slowest. The apical part activated faster than the basal part in the bi-ventricles. (2) In the HF group, there was always a noticeable LV-RV AR gradient, with the LV activating more quickly than the RV. At 3 min of LDVF, the septum of the LV activated fastest, while the anterior activated the slowest. Similarly, the apical part activated faster than the basal parts in the bi-ventricles. (3) In both groups, the AR decreased dramatically for the first 90 s, after which it declined more smoothly. (4) The AR of both the RV and LV in the HF group were slower than those in normal hearts for the first 90 s. From 90 s to the end of the analysis at 3 min of VF, neither the RV nor LV in HF hearts was activated differently from the bi-ventricles of the control animals.

### Possible mechanism of VF maintenance within 3 min

Data from previous animal studies suggest that one or two primary wavefronts located in the regions with the fastest AR drive the rest of the heart and that these regions, rather than the entire myocardium, are responsible for the maintenance of VF [12].

In the control group of this study, the overall wavefront direction of the bi-ventricles was from the apex to the base and from the posterior to the anterior at 3 min of LDVF. Previous studies have shown that the posterior LV activates faster than the anterior LV during VF in swine and that VF wavefronts tend to move from the posterior to the anterior. These reports are consistent with our findings [6, 13]. Therefore, if such a rotor is present, a possible site for a mother rotor is around the insertion of the posterior papillary muscle, which is located at the intersection of the posterior LV posterior wall and the posterior septum. This scenario may give rise to additional wavefronts that traverse the more slowly activating portion of the LV and the RV. Kim et al. [14] reported that the geometry of the ventricular wall and the anatomical structures, such as the posterior papillary muscle, influences the wave breaks and the maintenance of VF. Moreover, Pak et al. [15] demonstrated that ablation targeting the posterior papillary muscle reduces the potential for induction of VF, suggesting that eliminating the anchoring site might prevent sustained reentry and VF. The VF AR differences between the fastest and slowest regions in the present study were not large; Zaitsev et al. [16] and Samie et al. [17] reported larger differences in the AR between the fastest and slowest regions. Therefore, it is not known if these smaller AR differences are sufficient to support the mother rotor hypothesis. However, in human VF, rotors are not stable temporally and spatially over greater surfaces; these high-frequency regions thus require further study regarding VF maintenance.

In the HF group at the 3 min LDVF, we demonstrated that the AR of RV was much slower than the LV and that the septum of the LV activated more rapidly than any other portion of the LV or the RV, indicating that if there existed a mother rotor, it was probably located in the septum. This hypothesis is supported by the findings of Ikeda et al., who studied endocardial activation patterns during VF, in which the septum was found to have more wavelet numbers and a shorter cycle length than the other portions of the LV and RV [18]. It has been proposed that a smaller critical mass is required to maintain VF in the septum. All these observations suggest the possibility that the septum may serve as a possible source for the dominant region during VF in HF canines.

### Inter-ventricular differences in the endocardial AR

Rogers et al. [19] described the activation differences between the RV and LV during VF in normal porcine hearts, suggesting that there is a quantifiable difference between the LV regions, showing more wavefronts and activations than the RV. Umapathy et al. reported that the LV has a larger dominant frequency span than the RV [5]. The presence of spatially distributed gradients in the density of the inward rectifier current (I_k1_), with the left ventricular myocytes showing significantly weaker inward-directed rectification than the right ventricular myocytes, has been postulated to play crucial roles in the LV-RV gradients of excitation frequency during VF [20, 21]. In our study, we found that the AR of the LV differed from that of the RV for the first 20 s, but it did not show strong differences during the rest of the process. One possible explanation of the different findings is that the mapping methods have varied between different studies. For example, Rogers et al. recorded only parts of the epicardium, while in the present study, recordings were instead acquired via the global endocardial mapping of the two ventricles. Otherwise, since VF activations were not stable temporally and spatially over greater surfaces, these contradictory results might be explained by the spatial averaging of the excitation frequencies, which reduce the discriminatory characteristics between the regions.

In the HF dogs, there was always an AR gradient between the bi-ventricles as VF progressed. The gradient indicated that these regions participated in different ways in different animal models. For previous studies with diseased hearts, Huang et al. [11] demonstrated that VF in the setting of HF was significantly different from that in control dogs during VF. Their study showed that VF had a lower peak dV/dt, a slower AR, a significantly lower reentry occurrence, and an increased block occurrence relative to the VF in the controls. Moreno et al. [3] showed that the HF group had a lower dominant frequency and higher levels of organization during VF than in normal sheep hearts. Consistent with previous studies, the present study showed a slower AR in the HF animals. Everett et al. [22] showed that different ventricular substrates produced by different animal models altered the characteristics of VF. Thus, different mechanisms of VF might be present in the LV, which could contribute to the differences in the AR gradient distribution during VF between the normal and HF models.

### Intra-ventricular differences in the endocardial regional AR

The findings from our study demonstrated that there were regional differences in the different portions of the LV and RV, with some regions activating rapidly and other regions activating slowly. We observed a significant tendency for the VF wavefronts to spread from the apex toward the base of both the LV and RV in the normal and HF hearts. However, Nanthakumar et al. [4] did not find a significant direction from apex to base, suggesting that the epicardial base had the highest peak frequency. Earlier studies have revealed that there was no significant difference in the dominant frequency span between LV free wall and septum. In this study, the posterior wall of the LV activated the fastest in the control group, which seemed to coincide with the posterior papillary muscle. In the HF dogs, the septum of the LV activated the most rapidly, suggesting that the septum might be the source of the wave fronts. The differences in these results can be explained by differences in the species studied, differences in the heart sizes, differences in the animal models, and different effects between the isolated hearts and the intact hearts. From our analysis, the spatial differences in AR cannot be entirely explained by underlying fixed intrinsic anatomical changes or electrophysiological properties of the myocardium. It has been predicted that dynamic physiological factors may determine the regional frequency characteristics [2, 8]. The rapid dynamic increase in the wavefronts and other descriptors during VF may be independent of any underlying change to the intrinsic state of the heart.

### Clinical implications

A previous study reported that the incidence of out-of-hospital VF has declined compared with asystole as the initially recorded rhythm [23]. Cardiac pump failure without an arrhythmic event is suspected to be one of the factors. Although the AR of both the RV and the LV in the HF group were slower than those of normal hearts during the first 90 s interval, neither the RV nor the LV in the HF hearts were activated differently from the bi-ventricles in the control animals from 90 s to the end of analysis at 3 min of VF. Additionally, no LDVF episode was terminated within 3 min in HF hearts in the present study. Therefore, further study should be performed to detect whether LDVF in failing hearts is converted to asystole earlier than in normal hearts.

In the clinical setting, patients with preexisting HF are typically assumed to have a lower chance of successful cardiopulmonary resuscitation. The findings of this study indicate that LDVF is maintained differently in HF hearts than it is in normal hearts. Various intra- and inter-ventricular areas were activated differently during LDVF under the condition of HF. Some areas might be triggered by early or delayed after-depolarization with increased AR. Thus, in addition to electrical shock, other treatments, including agents that inhibit triggered activities and pacing, could potentially improve the outcomes of cardiopulmonary resuscitation in such patients.

### Study limitations

The limitations of this study are as follows. First, although the global bi-ventricular endocardium was simultaneously mapped, our mapping technology could not detect the micro-reentry at the endocardium. The basket electrodes were widely spaced, such that the fine details of the endocardial activation sequence were not identified. Second, transmural mappings were not performed. Therefore, this study could not detect the presence of a transmural AR gradient between the epicardium and endocardium during VF.

## Conclusion

Estimates of the distribution of VF AR are not uniform across the endocardium. A global bi-ventricular endocardial AR gradient was observed in the first 20 s of SDVF but was not observed in LDVF in normal hearts. However, the AR gradient was always present in both SDVF and LDVF in the HF hearts. The findings of this study suggest that the LDVF in the HF hearts is maintained differently from normal hearts, which accordingly leads to different recommended management strategies during LDVF resuscitation.

## Abbreviations

AR: Activation rate
FFT: Fast Fourier transform
HF: Heart failure
LDVF: Long-duration ventricular fibrillation
LV: Left ventricle
LV-H: Left ventricle in the HF group
LV-N: Left ventricle in the normal group
RV: Right ventricle
RV-H: Right ventricle in the HF group
RV-N: Right ventricle in the normal group
SDVF: Short-duration ventricular fibrillation
VF: Ventricular fibrillation
